# Community-Associated Methicillin-Resistant *Staphylococcus Aureus* Colonization in a Birth Cohort of Early Childhood: The Role of Maternal Carriage

**DOI:** 10.3389/fmed.2021.738724

**Published:** 2021-10-26

**Authors:** Ming-Han Tsai, Chih-Yung Chiu, Kuan-Wen Su, Sui-Ling Liao, Hsiang-Ju Shih, Man-Chin Hua, Tsung-Chieh Yao, Shen-Hao Lai, Kuo-Wei Yeh, Li-Chen Chen, Jing-Long Huang

**Affiliations:** ^1^Department of Pediatrics, Chang Gung Memorial Hospital, Keelung, Taiwan; ^2^Chang Gung University College of Medicine, Taoyuan, Taiwan; ^3^Molecular Infectious Disease Research Center, Chang Gung Memorial Hospital, Taoyuan, Taiwan; ^4^Division of Pulmonology, Department of Pediatrics, Chang Gung Children's Hospital, Taoyuan, Taiwan; ^5^Division of Allergy, Asthma, and Rheumatology, Department of Pediatrics, Chang Gung Children's Hospital, Taoyuan, Taiwan; ^6^Department of Pediatrics, New Taipei Municipal TuCheng Hospital, Chang Gung Memorial Hospital and Chang Gung University, New Taipei, Taiwan

**Keywords:** methicillin-resistant *Staphylococcus aureus*, children, mother, colonization, smoking

## Abstract

**Background:** Methicillin-resistant *Staphylococcus aureus* (MRSA) colonization in infants may pose a risk for subsequent infection in children. The study aimed to determine *S. aureus* colonization patterns in infancy, and strain relatedness between maternal and infant colonization.

**Methods:** A prospective cohort study was conducted for nasopharyngeal *S. aureus* detection in neonates at delivery; in children at 1, 6, 12, 24, 36, and 60 months of age; and from mothers immediately after the delivery of their baby and when their child is 1 month old. A questionnaire for infants and mothers was administered at each planned visit.

**Results:** In total, 521 and 135 infant–mother dyads underwent nasopharyngeal swab collection at 1 month and immediately after delivery, respectively. Among the 521 dyads at 1 month of age, concordant *S. aureus* colonization was found in 95 dyads, including MRSA in 48.4% (46/95). No concordant MRSA carriage was present among the 135 dyads at delivery. The genetic relatedness of concurrent MRSA-colonized dyads showed that more than two-thirds (32/46 [69.6%]) had identical genotypes, mainly ST 59/PVL-negative/SCC*mec* IV. Infants aged 1 month had the highest incidence of *S. aureus*, and the trend declined to a nadir at the age of 12 months. Carrier mothers who smoked cigarettes may increase the risk of infant *Staphylococcus* colonization (odds ratio, 2.12; 95% confidence interval, 1.23–3.66; *p* < 0.01).

**Conclusions:** Maternal–infant horizontal transmission may be the primary source of MRSA acquisition in early infancy. The avoidance of passive smoking could be recommended for the prevention of *S. aureus* carriage.

## Introduction

*Staphylococcus aureus* is a major cause of infectious diseases ranging from soft-tissue infections to bacteraemia, which causes substantial morbidity or mortality in hospital settings and communities (1, 2). Methicillin-resistant *S. aureus* (MRSA) is usually considered a hospital pathogen, and its infection has traditionally been confined to individuals with health care–associated factors, including recent hospitalization, residence in long-term care units, indwelling catheters, or haemodialysis (3). In the past decades, the prevalence of infections caused by MRSA has increased among healthy people, particularly among children without established risk factors for MRSA acquisition (4), namely, community-associated MRSA (CA-MRSA).

In Taiwan, CA-MRSA infections have been increasingly reported in children since 2002, and the proportion of MRSA infections to the community-associated *S. aureus* infections in children without risk factors increased from 9.8% during 1997–2000 to 56% in 2004–2005 (5). Chen et al. (4) showed that MRSA has accounted for 50% of childhood community-associated *S. aureus* infections in Taiwan since 2005. In addition, the burden of *S. aureus* infection in infants is high. In the United States, hospitalized infants under 1 yr of age had the highest risk of *Staphylococcus* infection, highest annual incidence, and highest mortality among children under 17 yr of age (6). Although a high incidence of childhood CA-MRSA infection has occurred in the past decade, the cause of this increase is not well understood.

Colonization by *S. aureus* may play an important role in the development of *Staphylococcus* infection (7). Previous studies have shown that MRSA colonization poses a significantly greater risk to the development of subsequent infection than methicillin-sensitive *S. aureus* (MSSA) colonization (8). To understand the increasing rate of CA-MRSA infections in children, an investigation on the timing and source of MRSA acquisition in infants is required. Maternal MRSA carriage may also play a role in neonatal or infantile colonization (9); however, the phenotypic and molecular features of *S. aureus* and MRSA isolates between mothers and infants have rarely been assessed.

A birth cohort study was conducted, and infants were prospectively examined for colonized *S. aureus* at the planned time points. The dynamics of *S. aureus* carriage during early childhood, the possible factors involved in MRSA colonization, and the molecular relationships between maternal and childhood *S. aureus* carriage were investigated.

## Methods

### Study Population

An ongoing prospective study launched in 2012 that follows a birth cohort of infants, was initiated in Keelung Chang Gung Memorial Hospital to investigate bacterial colonization and factors related to the development of asthma and other allergic diseases. Keelung Chang Gung Memorial Hospital, which is a district hospital with a capacity of 1,100 beds in Keelung, Taiwan, is capable of handling academic research, clinical services, and medical education. Women admitted to the obstetrics clinic of this hospital during the third trimester of pregnancy were invited to participate in the study. The study project was approved by the institutional review board of Keelung Chang Gung Memorial Hospital, and written informed consent was obtained from each participant.

### Study Design and Sample Collection

From March 2013 to December 2019, the mothers were requested to bring the enrolled infants to the pediatric clinic of Keelung Chang Gung Memorial Hospital for *S. aureus* detection from nasopharyngeal swabs at 1, 6, 12, 24, 36, and 60 months of age. One swab per child was collected per visit. A nasopharyngeal swab was collected from the mother when the swab was taken from the infant at the age of one month. To further investigate maternal and infantile carrier status at delivery, nasopharyngeal swabs were collected from the mothers and infants on the day of delivery starting from March 2015. Additionally, vaginal group B *Streptococcus* (GBS) screening was performed between gestational weeks 35 and 37 during routine maternal prenatal examination, and samples from the volunteer pregnant women were collected for *S. aureus* detection during the same period. Questionnaires were administered at each planned visit to obtain information such as demographic data, housing, living conditions, risk factors for *Staphylococcus* carriage, history of *Staphylococcus* infection and hospitalization, and other medical illnesses.

### Identification and Characterization of *S. aureus*

Nasopharyngeal or vaginal swabs were collected using separate cotton-tipped swabs (Copan Swab Applicator, Copan Diagnostics Inc., Brescia, Italy) at the scheduled visits. After being placed in a transported medium, swabs were transported to the microbiology laboratory within two h after collection and were cultured for bacteria by using standard methods for identification (10). Swab samples were inoculated onto trypticase soy agar with 5% sheep blood plates by using the streak plate method. On the basis of the patterns of beta*-*haemolysis and the macroscopic appearance, colonies of suspected *S. aureus* were further incubated on 5% sheep blood agar plates at 37 °C overnight. After incubation, a coagulase test was performed using rabbit plasma to identify *S. aureus*. To detect MRSA in specimens, *S. aureus* was first identified by a coagulase test, and a cefoxitin test was conducted to distinguish MRSA from MSSA in accordance with the recommendations of CLSI document M100-S18 (11). All *S. aureus* isolates were stored for further molecular characterization.

Pulsed-field gel electrophoresis (PFGE) with SmaI digestion was performed according to previously described procedures (12), and the genotypes were designated in alphabetical order. PFGE patterns with fewer than four band differences from an existing genotype were defined as subtypes of this genotype (13). Two isolates were considered indistinguishable, highly related, or distinct if they had the same subtype (no band difference), the same genotype (less than four band differences), or different types (equal to or greater than four band differences). Isolates with representative PFGE patterns were selected, and multilocus sequence typing (MLST) was conducted (http://www.mlst.net) (14). The allelic profiles were assigned by comparing the sequence at each locus with the sequences of the known alleles in the *S. aureus* MLST database, and the resulting profiles were defined as sequence types (STs) accordingly.

The *Staphylococcus* cassette chromosome (SCC*mec*) typing of isolates was determined using multiplex PCR, as described previously (15). The control strains for SCC*mec* types I, II, III, and IVa were as follows: type I, NCTC10442; type II, N315; type III, 85/2082; and type IVa, JCSC4744. SCC*mec* typing for type V_T_ was determined using a specific primer described elsewhere (13), and the TSGH-17 strain was used as a control. The presence of Panton–Valentine leucocidin (PVL) genes was determined by PCR, as described by Lina et al. (16).

### Statistical Analysis

Data were analyzed using SPSS (version 22.0; SPSS Inc., Chicago, IL, USA). Student's *t*-test was used to analyse the numerical data. If the data were not normally distributed, the Mann–Whitney U test was used for analysis. The chi-square test or Fisher's exact test was used when appropriate to analyzed the categorical data. In univariate analysis, clinical variables were compared between carrier and non-carrier mothers if their infants were *S. aureus* carriers at the same time. Variables for which *P* < 0.1 were chosen for model selection in multivariate analysis. Statistical significance was set at *P* < 0.05.

## Results

### Study Population

**Figure 1:**
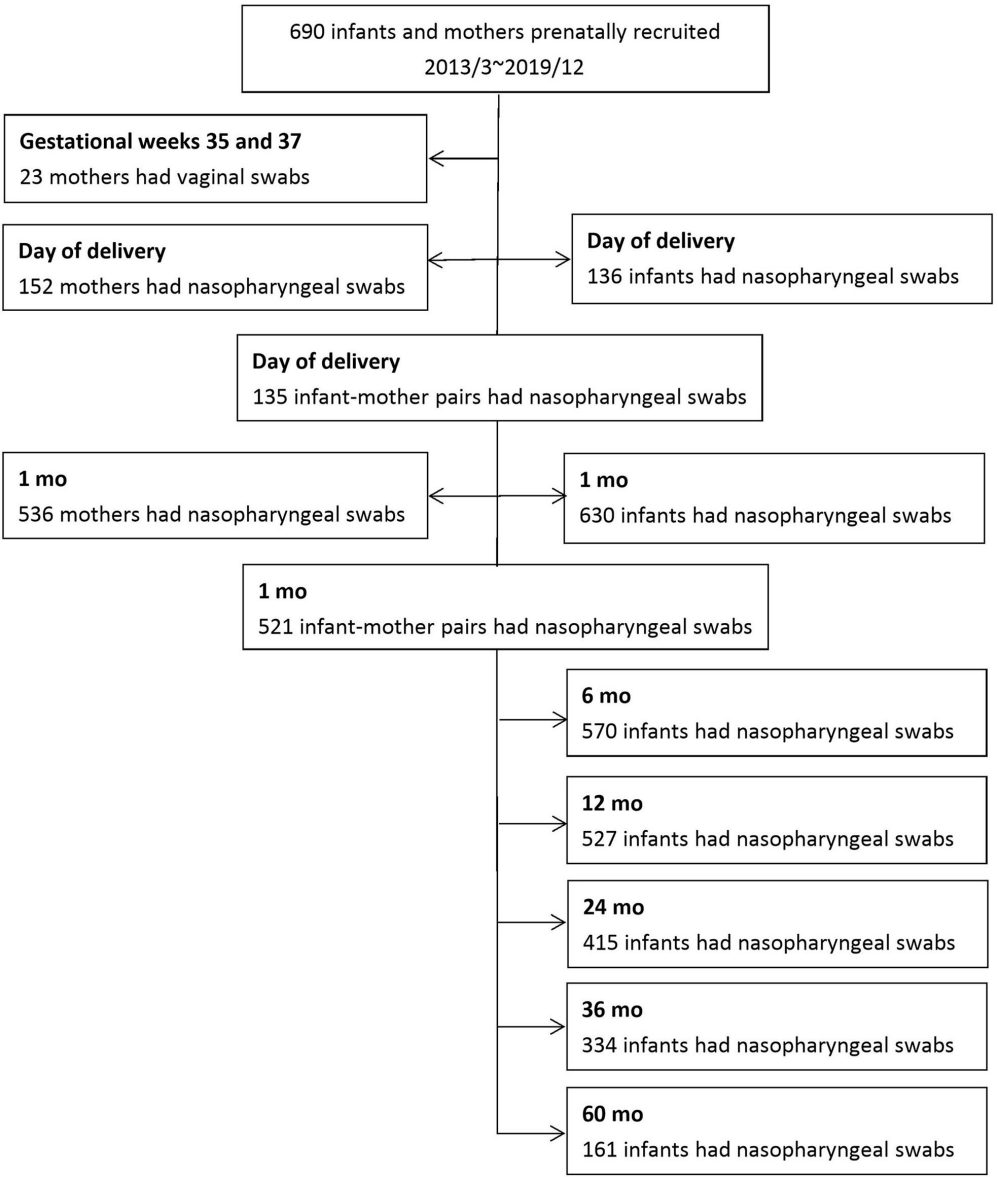
Flowchart of enrollment during the study period.

From March 2013 to December 2019, 690 infants and their mothers were prenatally recruited. Questionnaire-based surveys and nasopharyngeal swabs were received from 630 infants and 536 mothers one month past delivery. Since March 2015, nasopharyngeal swabs were collected from 136 infants and 152 mothers on the day of delivery. In total, 521 infant–mother pairs were enrolled one month past delivery, and 135 dyads were collected at delivery. Vaginal swabs from 23 volunteer pregnant women were obtained when routine GBS cultures were performed between gestational weeks 35 and 37. Figure 1 shows the detailed enrollment of this cohort.

**Figure 2:**
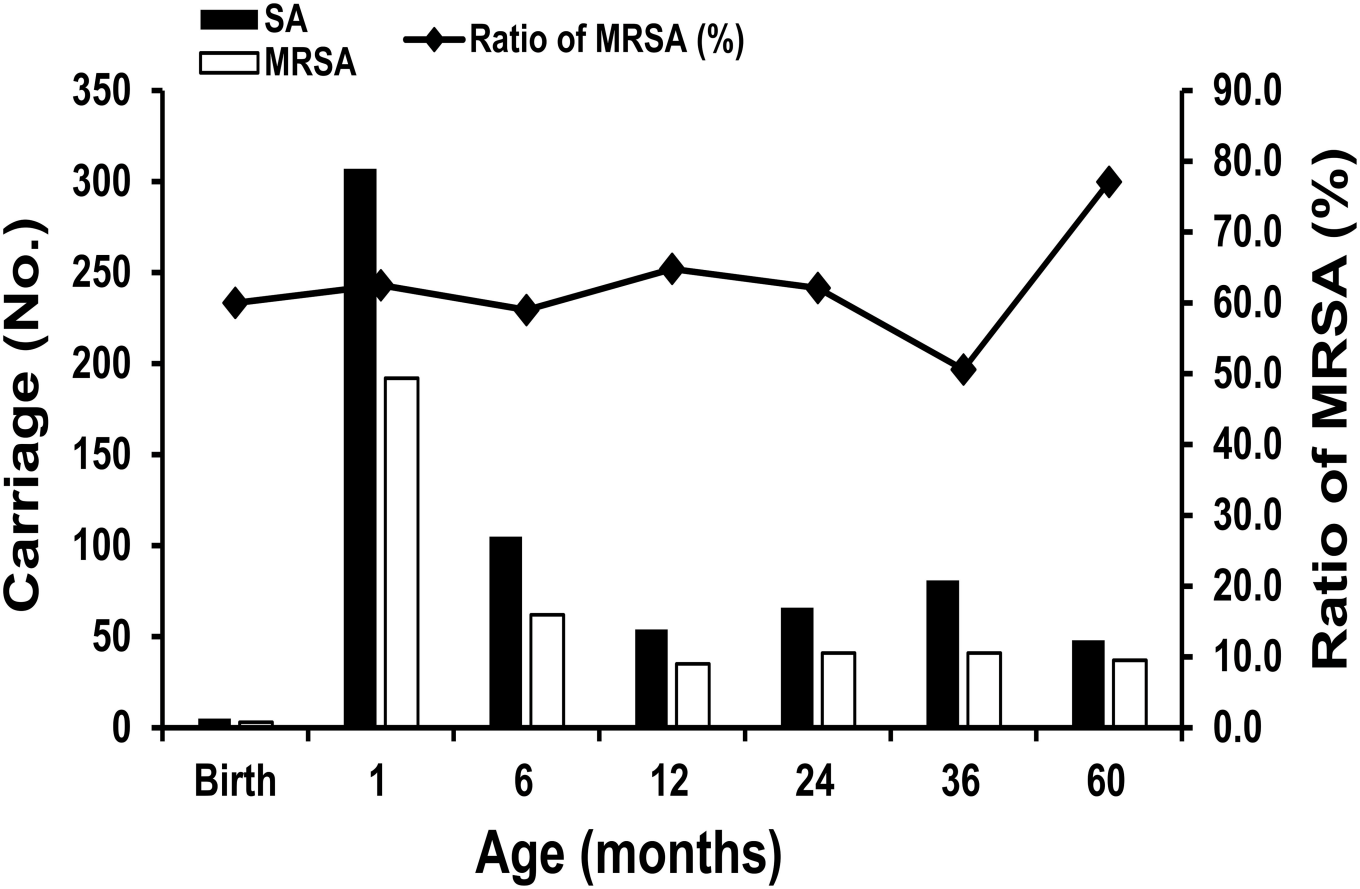
The trend of *Staphylococcus aureus* colonization, including methicillin-resistant *Staphylococcus aureus* (MRSA) at the planned sampling time points during the first 60 months of life.

Regarding the *S. aureus* carriage in children during the first 60 months of life, *S. aureus* colonization was observed in 3.7% (5/136) at delivery, 48.7% (307/630) at 1 month of age, 18.4% (105/570) at 6 months of age, 10.2% (54/527) at 12 months of age, 15.9% (66/415) at 24 months of age, 24.3% (81/334) at 36 months of age, and 29.8% (48/161) at 60 months of age. MRSA colonization was noted in 2.2% (3/136) at birth, 30.5% (192/630) at 1 month, 10.9% (62/570) at 6 months, 6.6% (35/527) at 12 months, 9.9% (41/415) at 24 months, 12.3% (41/334) at 36 months, and 23.0% (37/161) at 60 months. Infants aged 1 month had the highest incidence of *S. aureus* and MRSA carriage, and the trend declined to a nadir at the age of 12 months. However, the carriage rate gradually increased between 24 and 60 months of age. Figure 2 shows the trend of *S. aureus* and MRSA colonization at the planned sampling time points during the first 60 months of life.

### Characterization of *S. aureus* Carriage and Genetic Relatedness Among Infant–Mother Pairs

Among the 521 dyads at the age of 1 month, *S. aureus* colonization was observed in 49.7% (259/521) of infants and 27.6% (144/521) of mothers. Concordant *S. aureus* colonization was found in 95 infant–mother dyads, including MRSA in 48.4% (46/95), MSSA in 33.7% (32/95), and mixed MRSA/MSSA in 17.9% (17/95). Additionally, 3.7% (5/135) colonized infants and 23.7% (32/135) mothers were noted among the 135 dyads at delivery. However, only one pair of concordant *S. aureus* carriage was found among these dyads, and the *S. aureus* isolates belonged to MSSA.

For the molecular investigation of concordant *S. aureus*–colonized isolates, 95 infant–mother pairs at 1 month of age were obtained to analyze strain relatedness. The results revealed that 56.8% (54/95) had identical genotypes, including 69.6% (32/46) concordant MRSA [mainly ST 59/PVL-negative/ SCC*mec* IV, 54.3% (25/46)], 56.3% (18/32) concordant MSSA, and 23.5% (4/17) mixed MRSA/MSSA colonized pairs belonging to genetically indistinguishable genotypes. Table 1 and Table S1 show the molecular characteristics of *S. aureus* isolates among infant–mother pairs.

**Table 1:** Molecular relatedness of concordant colonization with *Staphylococcus aureus* in infant-mother pairs.

	**Concordant** ***S. aureus***		**Concordant MRSA**		**Concordant MSSA**		**Concordant mixed MRSA/MSSA**	
	**Concordant** **colonization**	**Genetic^**a**^** **similarity**	**Concordant** **colonization**	**Genetic^**a**^** **similarity**	**Concordant** **colonization**	**Genetic^**a**^** **similarity**	**Concordant** **colonization**	**Genetic^**a**^** **similarity**
Delivery^b^	1	0			1	0		
1 mo^c^	95	54 (56.8%)	46	32 (69.6%)	32	18 (56.3%)	17	4 (23.5%)

*S. aureus, Staphylococcus aureus; MRSA, methicillin-resistant Staphylococcus aureus; MSSA, methicillin-sensitive Staphylococcus aureus*.

a *Genetic similarity denotes isolates from infant-mother pairs were of indistinguishable genotypes*.

b *Number of infant-mother pairs at delivery was 135, and only one pair of concordant Staphylococcus aureus colonization was found among the 135 dyads*.

c *Number of infant-mother pairs at the age of 1 month was 521, and concordant Staphylococcus aureus colonization was found in 95 of them*.

Among the 135 dyads at delivery, nasopharyngeal swabs were also administered for 124 dyads at 1 month of age. To longitudinally investigate the association between infant and mother carriers, the *S. aureus* characteristics of the 124 infant–mother pairs were further analyzed. Among these dyads, *S. aureus* colonization was observed in 1 infant (MSSA) and 31 mothers at delivery, but no concordant *S. aureus* carriage was present. In addition, 51.6% (64/124) of infants and 25.8% (32/124) of mothers were colonized at the age of 1 month, and concordant *S. aureus* colonization was found in 25 dyads, including MRSA in 52.0% (13/25), MSSA in 32.0% (8/25), and mixed MRSA/MSSA in 16.0% (4/25). With regard to the investigation of molecular genotype, most of the concordant MRSA-colonized dyads (69.2%, 9/13) were genetically indistinguishable genotypes, followed by 37.5% (3/8) with identical genotypes in MSSA dyads and 25.0% (1/4) in mixed MRSA/MSSA dyads.

Twenty-three mothers had vaginal *S. aureus* colonization during pregnancy. Among the 23 mothers with vaginal *S. aureus* colonization, 6 had nasopharyngeal colonization at the age of 1 month. Concordant *S. aureus* infant carriers were found among them, including MSSA in four mothers, MRSA in one mother, and mixed MRSA/MSSA in one mother. The results of the genetic relatedness of concordant strains from mothers with vaginal *S. aureus* colonization, mothers with nasopharyngeal *S. aureus* colonization, and infants with nasopharyngeal *S. aureus* colonization showed that most of them (83.3%, 5/6) had identical genotypes (ST 59/pulsotype A1/PVL-negative/ SCC*mec* IV), and all infants were born with vaginal delivery.

### Comparison of Clinical Features Between Colonized Infants With Carrier Mothers and Without Carrier Mothers

To understand the influence of maternal carriage on infant *S. aureus* colonization, three variables, including demographic features, environmental factors, and health conditions, were compared between colonized infants with carrier mothers and those with non-carrier mothers (Table 2). Except for passive smoking exposure (OR 2.06, 95% CI 1.22–3.47, *P* < 0.01), no other variables were significantly associated with infant colonization with *S. aureus* in the univariate logistic regression analysis. Furthermore, passive smoking exposure related to infant *S. aureus* colonization was also found after multiple logistic regression analysis (OR 2.12, 95% CI 1.23–3.66, *P* < 0.01). Infant exposed to burning incense lighted by the carrier mothers seemed to be negatively associated with *S. aureus* colonization, but the difference was not statistically significant (OR 0.61, 95% CI 0.36–1.05, *P* = 0.08).

**Table 2:** Univariate and multivariate analyses of clinical features of infants and mothers by maternal *Staphylococcus aureus* carrier status.

**Variables**	**Value**		**Odds ratio** **(95% confidence interval)**	***p*-value**
	**Cases with maternal *S. aureus* colonization** ***N* = 95**	**Cases without** **maternal *S. aureus* colonization** ***N* = 164**		
**Factors associated with** ***Staphylococcus aureus*** **colonization of infants by univariate logistic regression analysis**				
**Demographics**				
Male (%)	50 (52.6)	86 (52.4)	0.99 (0.60–1.65)	0.98
**Breastfeeding** ^*^				
Breastfeeding (%) since birth	81/89 (91.0)	143/160 (89.4)	0.83 (0.34–2.01)	0.68
Breastfeeding period (mo) median (interquartile range)	6.0 (1.0–12.0)	4.0 (1.0–9.8)	1.04 (0.99–1.10)	0.13
Exclusive breastfeeding period (mo) median (interquartile range)	1.0 (0.0–10.5)	0.8 (0.0–6.0)	1.05 (1.00–1.11)	0.09
**Environmental**				
Passive smoking (%)	35/92 (38.0)	91/163 (55.8)	2.06 (1.22–3.47)	<0.01
No. of the family members median (interquartile range)	4.0 (3.0–5.0)	4.0 (3.0–6.0)	0.83 (0.61–1.13)	0.24
Moldy walls in houses (%)	41/93 (44.1)	70/162 (43.2)	0.97 (0.58–1.61)	0.89
Pet feeding during pregnancy (%)	27/93 (29.0)	51/162 (31.5)	1.12 (0.64–1.96)	0.68
Lighted incense in houses (%)	44/92 (47.8)	60/162 (37.0)	0.64 (0.38–1.08)	0.09
**Health conditions**				
PROM^†^ at birth (%)	2/92 (2.2)	11/162 (6.8)	3.28 (0.71–15.13)	0.13
Preterm birth (%)	11/94 (11.7)	18/164 (11.0)	0.93 (0.42–2.06)	0.86
Delivery via NSD (%)	64/94 (68.1)	103/164 (62.8)	1.26 (0.74–2.16)	0.39
PICU^‡^ admission at birth (%)	20/94 (21.3)	31/164 (18.9)	0.86 (0.46–1.62)	0.65
**Vitamin D deficiency**^**§**^ (%)				
Maternal Vitamin D deficiency	16/25 (64.0)	48/58 (82.8)	0.37 (0.13–1.07)	0.07
Infantile Vitamin D deficiency	16/23 (69.6)	39/55 (70.9)	0.94 (0.32–2.71)	0.91
Maternal chronic diseases^¶^	14/92 (15.2)	17/162 (10.5)	1.11 (0.55–2.25)	0.76
**Variables**		**Cases with maternal** ***S. aureus*** **carriage vs. without** ***S. aureus*** **carriage**		
		**Odds ratio (95% confidence interval)**		***p*** **-value**
**Factors associated with** ***Staphylococcus aureus*** **colonization of infants by multiple logistic regression analysis**				
Exclusive breastfeeding period (mo)		1.04 (0.98–1.10)		0.20
Passive smoking (%)		2.12 (1.23–3.66)		<0.01
Lighted incense in houses (%)		0.61 (0.36–1.05)		0.08
Maternal Vitamin D deficiency (%)		0.46 (0.15–1.43)		0.18

* *Infant with breastfeeding means that infant had a history of breastfeeding including exclusive breastfeeding for at least 4 wk*.

† *PROM indicates pre-labor rupture of membrane. It is defined as rupture of the membrane of the amniotic sac and chorion ≧1 h before the onset of labor*.

‡ *PICU indicates pediatric intensive care unit*.

§ *Vitamin D deficiency denotes serum 25-hydroxyvitamin D level <20 ng/mL*.

¶ *Maternal chronic diseases indicates mothers had chronic diseases including diabetic mellitus, hypertension, hyperthyroidism, or hypothyroidism*.

## Discussion

Our cohort showed that 10.2–48.7% and 6.6–30.5% of infants had *S. aureus* and MRSA carriage between and 2013–2019, respectively. The percentage was lower than that reported in other populations of Taiwanese children (*S. aureus* in 90%; MRSA in 40% of children aged <2 years) from 2005 to 2010 (14). The difference in the colonization rate may be attributed to the different study locations or periods. In the United States, a decline in the proportion of MRSA among *S. aureus* isolates (60.4% decrease) or a trend of MRSA infection (52% decrease) has been observed in the pediatric population since 2010 (17, 18).

Among the *S. aureus* carriage trends during the first five yr of life, the highest prevalence of *S. aureus* and MRSA carriage was observed at the age of one month, and the trend declined gradually in the first yr of life; however, the rising trend occurred between two and five yr of age. By contrast, an increasing trend of *Streptococcus pneumoniae* colonization was found during the first three yr of life in our previous report (19). This finding is consistent with those of other longitudinal studies conducted in Taiwan (20). The inverse relationship between *S. aureus* and *S. pneumoniae* might be explained by the pneumococcal inhibiting effect on *S. aureus*, which is mediated by the production of hydrogen peroxide by *S. pneumoniae* (21).

Results from our study showed that only 3.7% of infants had *S. aureus* colonization at delivery, and the colonization rate peaked (48.7%) in the first month of life. This finding supports our understanding that the fetus is sterile until shortly before birth and that the neonate becomes colonized with bacteria after birth (22). This suggests that infants acquire *S. aureus* mainly through horizontal transmission from surrounding carriers, possibly from family members. To clarify the association between maternal and infantile colonized isolates, the genetic relatedness of concurrent *S. aureus* and MRSA-colonized dyads was investigated. The results showed that 56.8% and 69.6% of concurrent *S. aureus* and MRSA-colonized dyads had identical genotypes, respectively. In the United States, Jimenez-Truque et al. (1) reported that 43% of *S. aureus*–colonized maternal–infant dyads had indistinguishable isolates, and Huang et al. (23) also found that half of the paired *S. aureus* isolates were genetically identical in their cohort. These results demonstrate the important role of maternal–infant horizontal transmission in *Staphylococcus* or MRSA carriage in early infancy.

Although the colonized infants might have acquired MRSA from their mothers via horizontal transmission, we cannot completely confirm this assumption if maternal colonization status before delivery is not analyzed. Thus, the association between the vaginal *Staphylococcus* colonization of mothers during pregnancy and nasopharyngeal carriage of infants after birth was investigated. One-fourth of infants (6/23 [26.1%]) born to mothers with vaginal *S. aureus* colonization had concurrent nasopharyngeal colonization. Although the number of maternal–infant pairs was small in our series, most of the concordant colonized isolates (5/6 [83.3%]) were genetically indistinguishable genotypes. This finding implies that vertical transmission may still occur in a minority of cases even though horizontal spread within family members (particularly from mother to infants) remains the primary method of MRSA acquisition in early infancy. Jimenez-Truque et al. (1) reported that infants born to mothers with *S*. *aureus* vaginal colonization were five times more likely to be nasally colonized within two h of birth. However, only 8.2% of pregnant women may be vaginally colonized with *S. aureus* according to the previous report (24). Therefore, the occurrence of vertical transmission is relatively uncommon and does not lead to a significant risk of *S. aureus* acquisition in early infancy.

Given the importance of maternal carriage on infantile *S. aureus* colonization, the clinical characteristics of infants and mothers according to maternal carrier status were compared. We analyzed demographic, environmental, and health-related factors being suggested to influence the infantile *S. aureus* carriage including gender, breastfeeding, number of family members, history of labor and environmental conditions (i.e., passive smoking or burning incense) (25). The result showed that infants born to smoking carrier mothers were found to be at risk of *S. aureus* colonization. This finding was consistent with those of previous studies which showed that passive smoking was associated with increased *S. aureus* colonization (26). The cause of passive smoke exposure associated with the carriage of pathogenic bacteria is not fully understood. One possible explanation is mucociliary transport alteration. Several studies have shown that cigarette smoke may reduce the ciliary beat of respiratory epithelial cells or impair respiratory epithelial ciliogenesis (27). Tobacco smoke may inhibit interleukin-8 and human β-defensin in sinonasal epithelial cell cultures derived from patients with chronic rhinosinusitis, thus suggesting that passive smoke may have a suppressive function on sinonasal innate immunity (28). Therefore, passive smoke exposure may compromise the antibacterial function of leukocytes and increase the risk of colonization by pathogenic *S. aureus* on respiratory epithelial cells. The influence of lighted incense on infantile *S. aureus* carriage was also investigated, but the result was not inconsistent with that of passive smoking. The cause of this inconsistency may be due to different toxicants present in incense and tobacco smoke resulting in differential cytotoxic or mutagenic effects on the bacterial community (29). Vallès et al. (30) found that the effect of burning incense exposure on microbiota composition may differ from those of tobacco exposure, and exposure to lighted incense was associated with depletion of the Firmicutes class *Bacilli* (e.g., *Staphylococcus*), which was compatible with our study result.

In our study, the most common clone accounting for 54.3% (25/46) of the MRSA isolates among infant–mother pairs were identified as ST 59/PVL-negative/SCC*mec* IV, namely, “Asian-Pacific” clone. A previous molecular epidemiology study of CA-MRSA in Taiwan revealed that two genotypes accounted for the majority of CA-MRSA strains, including the “Taiwan” clone (ST 59/PVL-positive/SCC*mec* V_T_) and an “Asian-Pacific” clone (ST 59/PVL-negative/SCC*mec* IV) (31). The isolates of both pulsotypes belonging to the ST 59 lineage had a similar genetic background; however, the Asian-Pacific clone was more prevalent in colonizing healthy individuals than the Taiwan clone (29). Among the MRSA carrier surveillance studies in 2001–2002 and 2005–2006, 62–78.3% of colonized isolates belonged to the Asian-Pacific clone, and these findings were similar to our study results (32, 33).

This study has some limitations. First, this is an ongoing project, but the period of this study was limited to 2013–2019; therefore, some enrolled cases could not complete the entire 60-month study course. Second, the number of pregnant women with vaginal *Staphylococcus* colonization was relatively small; therefore, the role of vertical transmission in infantile MRSA carriage should be interpreted with caution in our series.

In conclusion, most concordant MRSA-colonizing isolates were of indistinguishable genotypes, thus suggesting that maternal–infant horizontal transmission may be the primary source for MRSA acquisition in early infancy. Cigarette smoke may contribute to increased susceptibility to *Staphylococcus* colonization, and the avoidance of passive smoking could be recommended as an important strategy for the prevention of *S. aureus* carriage in infants.

## Data Availability Statement

The data analyzed in this study is subject to the following licenses/restrictions: Participant privacy prevents public sharing of individual-level data. Requests to access these datasets should be directed to H-JS, adashih4016@gmail.com.

## Ethics Statement

The studies involving human participants were reviewed and approved by the Institutional Review Board of Keelung Chang Gung Memorial Hospital, Taiwan. Written informed consent to participate in this study was provided by the participants' legal guardian/next of kin.

## Author Contributions

M-HT designed the study, collected and analyzed the data, and drafted and revised the manuscript. C-YC, K-WS, S-LL, M-CH, T-CY, S-HL, K-WY, and L-CC collected the data and reviewed the manuscript. H-JS did the experiment and collected the data. M-HT and J-LH reviewed and edited the manuscript. All authors contributed to the article and approved the submitted version.

## Funding

This study was supported by grants from Keelung Chang Gung Memorial Hospital, Taiwan (CMRPG2E0125 and CMRPG2K0311).

## Conflict of Interest

The authors declare that the research was conducted in the absence of any commercial or financial relationships that could be construed as a potential conflict of interest.

## Publisher's Note

All claims expressed in this article are solely those of the authors and do not necessarily represent those of their affiliated organizations, or those of the publisher, the editors and the reviewers. Any product that may be evaluated in this article, or claim that may be made by its manufacturer, is not guaranteed or endorsed by the publisher.

## Supplementary Material

Table S1 Molecular characteristics of methicillin-resistant *Staphylococcus aureus* isolates among 46 infant-mother pairs at the age of 1 month.
