# Supplementary material for: Community-Associated Methicillin-Resistant Staphylococcus Aureus Colonization in a Birth Cohort of Early Childhood: The Role of Maternal Carriage
Source: Front Med (Lausanne). 2021 Oct 26;8:738724. doi: 10.3389/fmed.2021.738724 (PMC8577750; doi:10.3389/fmed.2021.738724)
Supplement: Table S1 — Molecular characteristics of methicillin-resistant Staphylococcus aureus isolates among 46 infant-mother pairs at the age of 1 month. [file Table_1.docx]

**Table S1**

**Table S1. Molecular characteristics of methicillin-resistant *Staphylococcus aureus* isolates among 46 infant-mother pairs at the age of 1 month**

| **Case**  **No.** | **Characterization of MRSA isolates among infant-mother pairs at 1 month of age** | | | | | | | | | | |
| --- | --- | --- | --- | --- | --- | --- | --- | --- | --- | --- | --- |
|  | **Infants** | | | | |  | **Mothers** | | | | |
|  | PFGE | MLST | SCC*mec*  types | PVL  gene | mecA  gene |  | PFGE | MLST | SCC*mec*  types | PVL  gene | mecA  gene |
| 269 | A | 59 | IV | - | + |  | A | 59 | IV | - | + |
| 398 | A1 | 59 | IV | - | + |  | A2 | 59 | IV | - | + |
| 475 | A | 59 | IV | - | + |  | A | 59 | IV | - | + |
| 530 | H | 508 | IV | - | + |  | H | 508 | IV | - | + |
| 546 | B4 | 97 | cannot  be identified | - | + |  | B4 | 97 | cannot  be identified | - | + |
| 578 | C | 188 | cannot  be identified | - | + |  | A1 | 59 | IV | - | + |
| 582 | A2 | 59 | IV | - | + |  | A2 | 59 | IV | - | + |
| 594 | H2 | 508 | IV | - | + |  | H1 | 508 | IV | - | + |
| 598 | A1 | 59 | IV | - | + |  | H2 | 508 | IV | - | + |
| 603 | F | 59 | V_T_ | - | + |  | F | 59 | V_T_ | - | + |
| 616 | A1 | 59 | IV | - | + |  | A1 | 59 | IV | - | + |
| 618 | A1 | 59 | IV | - | + |  | A1 | 59 | IV | - | + |
| 630 | A1 | 59 | IV | - | + |  | A1 | 59 | IV | - | + |
| 642 | B | 15 | cannot  be identified | - | + |  | A1 | 59 | IV | - | + |
| 643 | A1 | 59 | IV | - | + |  | A1 | 59 | IV | - | + |
| 667 | A4 | 59 | IV | - | + |  | X | 45 | IV | - | + |
| 668 | A1 | 59 | IV | - |  |  | A1 | 59 | IV | - | + |
| 674 | H | 508 | IV | - | + |  | B2 | 15 | IV | - | + |
| 675 | B3 | 15 | cannot  be identified | - | + |  | A1 | 59 | IV | - | + |
| 677 | A4 | 59 | IV | - | + |  | A4 | 59 | IV | - | + |
| 680 | A | 59 | IV | - | + |  | C5 | 12 | IV | - | + |
| 682 | A | 59 | IV | - | + |  | H | 508 | cannot  be identified | - | + |
| 686 | A | 59 | IV | - | + |  | A | 59 | IV | - | + |
| 691 | A1 | 59 | IV | - | + |  | A1 | 59 | IV | - | + |
| 710 | A1 | 59 | IV | - | + |  | A1 | 59 | IV | - | + |
| 713 | A1 | 59 | IV | - | + |  | A1 | 59 | IV | - | + |
| 718 | A1 | 59 | IV | - | + |  | A1 | 59 | IV | - | + |
| 742 | A1 | 59 | IV | - | + |  | A1 | 59 | IV | - | + |
| 745 | A1 | 59 | IV | - | + |  | A1 | 59 | IV | - | + |
| 751 | A1 | 59 | IV | - | + |  | A1 | 59 | IV | - | + |
| 752 | A1 | 59 | IV | - | + |  | A1 | 59 | IV | - | + |
| 757 | A | 59 | IV | - | + |  | A | 59 | IV | - | + |
| 762 | H | 508 | IV | - | + |  | A5 | 59 | IV | - | + |
| 785 | C2 | 7 | IV | - | + |  | A1 | 59 | IV | - | + |
| 793 | I1 | 239 | IV | - | + |  | A6 | 59 | IV | - | + |
| 799 | A1 | 59 | IV | - | + |  | A1 | 59 | IV | - | + |
| 800 | B2 | 15 | IV | - | + |  | B2 | 15 | IV | - | + |
| 814 | A4 | 59 | IV | - | + |  | A4 | 59 | IV | - | + |
| 816 | A2 | 59 | V_T_ | - | + |  | A2 | 59 | V_T_ | - | + |
| 826 | A4 | 59 | IV | - | + |  | H | 508 | IV | - | + |
| 839 | A1 | 59 | IV | - | + |  | A1 | 59 | IV | - | + |
| 855 | A1 | 59 | IV | - | + |  | A1 | 59 | IV | - | + |
| 875 | A1 | 59 | V_T_ | + | + |  | L |  | cannot  be identified | - | + |
| 911 | C4 | 12 | V_T_ | + | + |  | A1 | 59 | V_T_ | + | + |
| 917 | A4 | 59 | IV | - | + |  | A4 | 59 | IV | - | + |
| 920 | A4 | 59 | IV | - | + |  | A4 | 59 | IV | - | + |

PFGE, pulsed-field gel electrophoresis; MLST, multilocus sequence typing; SCC*mec*, *Staphylococcus* cassette chromosome; PVL, Panton–Valentine leucocidin
